# Sexual initiation and associated factors among young women in West Shoa, Ambo Town, Ethiopia: a community-based cross-sectional study

**DOI:** 10.1186/s12905-018-0563-7

**Published:** 2018-05-30

**Authors:** Digafe Tsegaye Nigatu, Asefa Seme, Shewaye Fituma, Mesfin Tafa Segni

**Affiliations:** 1 Department of Public Health, College of Medicine & Health Sciences, Ambo University, Ambo, Ethiopia; 2 School of Public Health Addis Ababa University, Addis Ababa, Ethiopia; 3 Department of Public Health, College of Health Science, Arsi University, Assela, Ethiopia

## Abstract

**Background:**

For physiological as well as behavioral reasons, sexual debut increases young individuals’ risk for infection with sexually transmitted infection including HIV. It is fundamental to recognize the factors related to sexual debut in a broader context for designing and implementing effective interventions targeting youth.

**Methods:**

Community-based cross-sectional study was employed from January to May, 2013 among females of Ambo town. A multistage sampling technique was applied. The participants were selected using simple random sampling technique. Face to face interview using structured and pretested questionnaires were used to collect thedata from the study participants. Bivariate and multivariate logistic regression analysis was used to determine the predictors of sexual initiation.

**Results:**

Three hundred seventeen (49.9%) of the respondents have ever had sex. The mean age at first sexual initiation was 16.6 (SD ±2.3) years. Being in age group 20-24 [Adjusted Odds Ratio (AOR) & (95% CI) = 2.75 (1.74, 4.34)], Educational level [AOR& (95% CI) = 0.20 (0.08, 0.48)], being in school [AOR& (95% CI) = 0.19 (0.11, 0.33)], having paid job [AOR& (95% CI) = 2.20 (1.19, 4.07)], peer pressure [AOR& (95% CI) = 3.20 (2.08, 4.94)], alcohol consumption [AOR& (95% CI) = 2.17 (1.43, 3.28)], and pornographic materials [AOR& (95% CI) = 2.27 (1.43, 3.61)] had significant association with sexual initiation.

**Conclusion:**

Substantial numbers of females had started sexual activity that might expose them to different reproductive health problems. In general age group, peer pressure, alcohol consumption and watching pornographic materials were found to be predictors for the sexual debut. Therefore, building life skills, establishing youth friendly clubs should be intensified.

## Background

According to World Health Organization (WHO), youth is defined as an individual aged 15 to 24 years while adolescents are those aged 10 to 19 years and young people as those between the ages of 10 to 24 years old. Adolescence is a period of opportunity and risk when numerous young people practice critical and life-defining experiments such as their first sexual experience, marriage, pregnancy, and parenthood [1].

Worldwide, the youths aged 15 to 24 estimated for more than 1 billion people and the majority live in developing countries [2]. Youth represent slightly more than 20 % of Africa’s population.

In sub-Saharan Africa, coitalactivity is not a new phenomenon. In recent, maturity occurs at earlier age, and the time for marriage is increased; due to this fact the practice of sexual activity occur before marriage. Sexual debut increases youths’ risk for infection with numerous reproductive health issues including sexually transmitted infection and HIV [3, 4].

Ethiopia is one of the most fascinating countries in sub-Saharan Africa where there are diverse cultures, traditions, social values and practices that has impact on the sexual debut. Consequently, they become victims of reproductive health problems [5, 6].

Different literatures have demonstrated that the time of sexual initiation among female youths affected by socio-demographic factors and other factors such as pornographic materials, Khat and alcohol [7, 8]. Due to these effects, a substantial proportion of young people are engaged into sexual practice prematurely.

So far conducted studies demonstrated that the proportion of premarital sexual activity among 15–24 years young people in Oromia region was 31%, which is significantly higher than the national average (19%). However, factors that influence sexual initiation in Oromia youths have not yet been studied in detail [9].

Due to biological and cultural factors, girls are at a much greater risk at early ages than boys. In Ethiopia, young girls are more vulnerable to HIV than boys because of early age at sexual debut, early marriage, sexual abuse and violence such as rape and abduction [10]. Therefore, this study has been intended to determine the proportion of age at first sex and factors associated with sexual initiation among girls in the Ambo town.

## Methods

### Study design and study setting

Community-based cross-sectional study design was conducted among females in Ambo town from Jan. to May, 2013. Ambo town is the capital of West Shewa Zone, in Oromia regional state. The town located 114 km west of Addis Ababa and it has three *kebeles*. According to the town municipality, more than 67,514 populations live in the town in 2009 (2001 E.C) including the population of expansion areas; of which males accounts for 34,276 (50.8%) and females 33,238 (49.2%).

### Study participants & sample size calculation

The respondents were all female youths in the age group of 15 to 24, who lawfully reside in the town at the time of the survey. The sample size was considered by assuming a 95% level of confidence interval, 0.05 margins of error, and with expected prevalence of sexual initiation among female youths 51% [2]. Finally, by considering a design effect of 1.5 and 10% of non-response rate, the final sample size was 675.

### Sampling procedure

Finally, 675 respondents took part in the study. A multistage sampling procedure was employed. The town has three *kebeles* and two *kebeles* were selected by a lottery method. We then further subdivided these *kebeles* by Got (i.e village). From each Got/village, households were selected by simple random sampling, based on proportional allocation of the size of households in each Got or village and using the number of household as a sampling frame. The first households were selected from the town using the town’s house number registration by lottery method. In cases of selected household with more than one eligible study subject, only a single respondent was chosen by random method. In cases where no eligible respondent found in the chosen household, the data collectors have gone to the next household until they found an eligible subject.

### Data collection & analysis

We adopted the questionnaire from Ethiopian Demographic Health Survey (EDHS) 2011 [6]. The questionnaire was used to elicit data from subjects on socio-demographic variables, sexual history, peer pressure, alcohol use, viewing pornographic materials which were independent variables in this study. The standardized questionnaire was first prepared in English. It was translated to Amharic and back to English again in order to maintain the validity of the instrument. Nine data collectors and one supervisor who were midwifery students from Ambo University, department of Midwifery were recruited. Data collectors and supervisors were trained for 1 day, before the pretest had been undertaken. To ensure the completeness and consistency of the collected data, supervisor and principal investigator had closely followed. Face to face interview using structured and pretested tool was used.

Epi Info Version 3.5.1 software was used to enter data. Then exported and analyzed using SPSS version 16.00. Descriptive statistical analysis with standard deviation was employed. To examine the potential predictors of sexual debut, bivariate and multivariate analysis was used. Variables which demonstrated *p* value less than 0.2 in the bivariate analysis were incorporated into multiple logistic regressions model for further analysis and *p*-values less than 0.05 indicated as statistically significance.

### Ethical issues

Ethical clearance was obtained from School of Public Health, research ethical review committee of Addis Ababa University (AAU). Formal letter of support was written to Ambo town health office by School of Public Health, AAU. To participate in this study, a written consent was obtained from respondents above 18 years old and from the parents/guardians for those whose age less than 18 years old females; after informing the objectives of the study; the right to participate and not participate or to terminate the interview at any time was fully explained before data collection. Respondents were free to decline participation, and we made it sure that if they decide so far administered data will not be stored. Confidentiality was maintained by not mentioning their name on the questionnaire.

## Results

### Socio-demographic variables

Three hundred fifty one (55.3%) were found in age group of 15-19 years. The mean age was 16.6 years (SD = 2.3, age range: 10-24). Three hundred ninety seven (62.7%) were Oromo in ethnicity. Orthodox Christian followers (51.9%) were taken the lead in the study area. Regarding marital status of the respondents, 80.0% were singles or unmarried. Two hundred forty seven (38.9%) were preparatory and TEVT and 74.8% were attended in school at the time of the survey (Table 1).

**Table 1:** Socio-demographic characteristics of the respondents in Ambo, April 2013

Variables	Number	Percent (%)
Age group (years)		
15–19	351	55.3
20–24	284	44.7
Ethnicity		
Oromo	397	62.5
Amhara	144	22.7
Tigre	58	9.1
Others	36	5.7
Religion		
Orthodox	330	51.9
Protestant	193	30.4
Muslim	65	10.2
Catholic	32	5.0
Others	15	2.4
Marital status		
Married	127	20.0
Unmarried	508	80.0
Education		
Primary school 1–8	112	17.6
Secondary school 9–10	222	35.0
Preparatory & TEVT	247	38.9
College & Above	54	8.5
Currently in school		
Yes	475	74.8
No	160	25.2

### Sexual history

Three hundred seventeen (49.9%) of the surveyed youths have ever had sex. The Mean & median age of sexual initiation in this study were 16.6 years (SD = 2.3) (Range 10–24 years). Three-fifth (60%) of the respondents was initiated sex before age of 18 years. The main reasons for engaging in first time sex were passionate love (30.7%), any substance use (25.7%), feeling maturity (18.7) and followed by desire for marriage (12.6%) (Table 2).

**Table 2:** Reasons to start sex by respondents in Ambo town; Ethiopia; 2013

Variables	Number	Percent (%)
Passionate love	195	30.7
Any substance use	163	25.7
Feeling maturity	124	18.67
Desire for marriage	80	12.6
Peer pressure	39	5.87
Rape	38	5.72
Receiving gift	13	1.96
Pornographic material	12	1.9

### Peer influence

The role of peer pressure on sexual debut was observed in this study. Two hundred thirty one (36.4%) were responded that they had ever been encouraged by their friends to take part in sexual activities. Furthermore, 24.9% of respondents had been encountered pressure from their peers to have sexual intercourse more frequently (Table 3).

**Table 3:** Peer pressure and non-sexual related behaviors regarding sexual debut among female youths in Ambo town; Ethiopia April 2013

Variables	Frequency	Percent
Had sex		
Yes	317	49.9
No	318	50.1
Peer pressure		
Yes	231	36.4
No	404	63.6
Peers influence to have sexual intercourse		
Frequently	158	24.9
Occasionally	243	38.3
Not at all	234	36.8
Used any substance		
Yes	193	30.4
No	442	69.6
Drunk alcohol		
Yes	299	47.1
No	336	53.9
Viewed Pornographic material		
Yes	234	36.8
No	401	64.2

### Behaviors of non sexual related risks

One hundred ninety three (30.4%) have used substances. The most abused substance was Khat (94.6%).respondents were also practiced other non sexual risk behaviors such as drinking alcohol 299 (47.1%), viewing pornographic materials 234 (36.2%).

### Factors associated with sexual debut

Educational status, peer influence, age, any substance use, being in school, drinking alcohol and viewed pornographic materials had showed statistically significant (*p*-value < 0.05) association with sexual debut in bivariate logistic regression analyses. After controlling for potential confounding factors, logistic regression analysis showed that, those young women who chewed Khat were found to 1.3 times more likely to already have been engaged to sexual intercourse than those who did not consume Khat”). Furthermore, those who drank alcohol and viewing pornographic materials were found to 2.2 times and 2.3 times more likely to initiate sexual activities respectively (Table 4).

**Table 4:** Factors contributing to sexual initiation among female youths, in Ambo town, April 2013, Ethiopia

Characteristics	Sexual Initiation		Crude OR (95% CI)	Adjusted OR (95% CI)
	Yes	No		
Age group (years)				
15–19	104 (33.7)	236 (72.4)	1.00	1.00
20–24	205 (66.3)	90 (27.6)	5.17 (3.68, 7.25)	2.75 (1.74, 4.34)
Education				
Primary school 1–8	77 (24.9)	105 (32.2)	0.15 (0.06, 0.31)	0.25 (0.10, 0.62)
Secondary school 9–10	94 (30.4)	128 (39.3)	0.14 (0.06, 0.31)	0.20 (0.08, 0.48)
Preparatory & TEVT	93 (30.1)	84 (25.8)	0.22 (0.10, 0.48)	0.30 (0.12, 0.73)
College & Above	45 (14.6)	9 (2.8)	1.00	1.00
Currently in school				
Yes	151 (48.9)	284 (87.1)	0.14 (0.09, 0.21)	0.19 (0.11, 0.33)
No	158 (51.1)	42 (12.9)	1.00	1.00
Peer influence				
Yes	163 (52.8)	86 (26.4)	2.92 (2.09, 4.08)	3.20 (2.08, 4.94)
No	146 (47.2)	240 (73.6)	1.00	1.00
Used any substances				
Yes	98 (31.7)	73 (22.4)	10.1 (3.94, 25.89)	1.30 (0.83, 2.06)
No	211 (68.3)	253 (77.6)	1.00	1.00
Drank alcohol				
Yes	180 (58.3)	109 (33.4)	8.26 (5.25, 13.0)	2.17 (1.43, 3.28)
No	129 (41.7)	217 (66.6)	1.00	1.00
Viewed pornographic materials				
Yes	144 (46.6)	73 (22.4)	3.46 (2.43, 4.92)	2.27 (1.43, 3.61)
No	165 (53.4)	253 (77.6)	1.00	1.00

Significance: *p* < 0.05., 1.00: Reference category

## Discussion

We have found that 49.9% have ever had sex. The finding is similar to other studies conducted in different countries on sexual behavior among female youths. The mean age at first sexual debut was 16.6 (+SD = 2.3) years and this is comparable with other previous studies in Ethiopia [2, 11]. However, the mean year of sexual debut in this study is higher than previous studies conducted in Ethiopia, Ethiopian Demographic Health Survey (EDHS) 2016 (16.6 years), in Dessie, north east Ethiopia (16.8 years), in Kolladiba, north west Ethiopia (15 years), in Gojam, north west Ethiopia (13.5 years) and in Butajira, southern Ethiopia (16 years) [2, 12–14]. The difference might be due to the decrease in early marriage [15] and because of the recently endorsed family law [16].

We have found that 231 (36.4%) had ever been encouraged by their friends to practice sexual activities. Furthermore, 24.9% of respondents had been encountered pressure from their peers to have sexual intercourse regularly. Furthermore, logistic regression analysis also revealed that respondents’ external pressure lead to first sex, which is similar with a cross-sectional and the National Longitudinal Study of Adolescent Health conducted in three Developing Countries, University of Minnesota, Minneapolis and Penn Institute for Economic Research [17], which revealed that peer norms have a substantial effect on the timing of sexual initiation for both boys and girls. According to Social-psychological theories of health behavior, adolescents’ sexual behaviors are influenced by the sexual attitudes and behaviors of their friends [18]. As different studies demonstrated, adolescents who are highly involved with their friends may find themselves in social contexts that encourage early dating and entry into romantic relationships, which have been linked to earlier sexual initiation.

Around 30 % of the respondents were used any substance to make feel high; mainly of Khat (94.6%), drinking alcohol 47.1% and viewing pornographic materials 36.8%. Illegal trading drug and misuse are now becoming more frequent in the country [19].

Our study finding demonstrated that substance users were found to be initiate sexual intercourse earlier than non-users, which is comparable with a cross-sectional study conducted in North East Ethiopia and nine European cities [2, 20].

A multivariate logistic regression analyses demonstrated that alcohol drinkers were twice at higher risk to initiate sexual intercourse than non-drinkers, which is similar with other findings [15, 21].

Viewing Pornographic material which typically followed by alcoholic drinks mentioned as a major contributing factor to sexual initiation in the study area. Pornographic material viewers were found to be twice more likely at higher risk to initiate sex, which is almost similar with a cross-sectional study done in Dessie, north east Ethiopia, revealed that the increased openness to Western culture has resulted in the invasion of pornographic videos, books, and magazines, whose consumers are mostly young people [2] and The National Longitudinal Study of Adolescent Health also supported this finding [22].

## Conclusions

The proportion of sexual debut was high; peer influences have a substantial effect on the timing of sexual debut for girls. Non sexual risky behaviors such as viewing pornographic materials, using any substances (such as Khat) and drinking alcohol especially at earlier age are independent predictors of sexual debut. Therefore, strengthening the norm of virginity, which delays sexual intercourse, should be advocated. There is need to target building life skills, establishing and strengthening youth friendly clubs, particularly for females, in making informed decisions about sexuality issues to reduce youth reproductive health problems. This intervention will certainly include provision of youth-friendly services by local government, non-governmental and community-based organizations.
